# Retraction Note: SWOT analysis of the application of three digital media in OLPE physical education teaching: Edmodo, Zoom, and Google Meet

**DOI:** 10.1186/s12909-026-09433-y

**Published:** 2026-05-15

**Authors:** Yongqi Gao, Li Zhu, Miao Tian

**Affiliations:** 1 College of Physical Education, Hubei Normal University, Huangshi City, Hubei Province 435002 People’s Republic of China; 2 School of Foreign Studies, Hubei University of Automotive Technology, Shiyan City, Hubei Province 442002 People’s Republic of China; 3 Organization Department, Hubei Normal University, Huangshi City, Hubei Province 435002 People’s Republic of China

**Retraction Note: BMC Medical Education 25, 243 (2025)**

**https://doi.org/10.1186/s12909-025-06826-3**

The Editor has retracted this article after concerns were raised regarding the accuracy of the references cited. Upon further investigation, it was observed that several references mentioned within the text were either not properly cited or missing from the reference list. Additionally, the article has unusually short methodology section. The Editor therefore no longer has confidence in the reliability of this article.

All authors disagree with this retraction.

